# The blockade of the TGF‐β pathway alleviates abnormal glucose and lipid metabolism of lipodystrophy not obesity

**DOI:** 10.1002/prp2.1160

**Published:** 2024-01-04

**Authors:** Wen‐Dong Xu, Shui‐Zheng Lai, Jia Zhao, Shi‐Jie Wei, Xue‐Ying Fang, Yi‐Yi Liu, Xiang‐Lu Rong, Jiao Guo

**Affiliations:** ^1^ Guangdong Metabolic Diseases Research Center of Integrated Chinese and Western Medicine Guangdong Pharmaceutical University Guangzhou China; ^2^ Key Laboratory of Glucolipid Metabolic Disorder, Ministry of Education of China Guangdong Pharmaceutical University Guangzhou China; ^3^ Institute of Chinese Medicine Guangdong Pharmaceutical University Guangzhou China; ^4^ Guangdong TCM Key Laboratory for Metabolic Diseases Guangdong Pharmaceutical University Guangzhou China

**Keywords:** adipose atrophy, adipose hypertrophy, adipose tissue, glucolipid metabolism, TGF‐β

## Abstract

TGF‐β is thought to be involved in the physiological functions of early organ development and pathological changes in substantial organ fibrosis, while studies around adipose tissue function and systemic disorders of glucolipid metabolism are still scarce. In this investigation, two animal models, aP2‐SREBP‐1c mice and ob/ob mice, were used. TGF‐β pathway showed up‐regulated in the inguinal white adipose tissue (iWAT) of the two models. SB431542, a TGF‐β inhibitor, successfully increased inguinal white adipocyte size by more than 1.5 times and decreased the weight of Peripheral organs including liver, Spleen and Kidney to 73.05%/62.18%/73.23% of pre‐administration weights. The iWAT showed elevated expression of GLUTs and lipases, followed by a recovery of circulation GLU, TG, NEFA, and GLYCEROL to the wild‐type levels in aP2‐SREBP‐1c mice. In contrast, TGF‐β inhibition did not have similar effects on that of ob/ob mice. In vitro, TGF‐β blocker treated mature adipocytes had considerably higher levels of glycerol and triglycerides than the control group, whereas GLUTs and lipases expression levels were unchanged. These findings show that inhibiting the abnormally upregulated TGF‐β pathway will only restore iWAT expansion and ameliorate the global metabolic malfunction of glucose and lipids in lipodystrophy, not obesity.

## Graphical abstract

TGF‐β is clinically overexpressed in both obesity and lipodystrophy WAT. Our study is the first time to demonstrate that inhibiting TGF‐β function improves iWAT function and normalizes systemic glucolipid metabolism. but did not significantly affect that of normal (3T3‐L1 cells) or over‐developed adipocytes (ob/ob mice). This discovery provides a better understanding of TGF‐β in adipose tissue in different pathological states and offers new strategies and ideas for the treatment of glucolipid metabolic disorders complicating adipose atrophy.

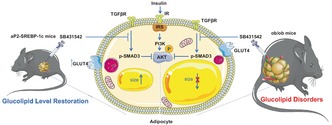

**Abbreviations**
AT: adipose tissue
ATGL: adipose triglyceride lipase
AUC: area under the curve
BAT: brown adipose tissue
cDNA: complimentary deoxyribonucleic acid
GLU: glucose
GLUT: glucose transporters
HDL: high‐density lipoprotein
HFD: high‐fat diet.
HSL: Hormone‐sensitive lipase
IR: insulin receptor
IRS: insulin receptor substrates
iWAT: inguinal white adipose tissue
MGL: monoglyceride lipase
NEFA: nonesterified fatty acid
OGTT: oral glucose tolerance test
PDE3B: phosphodiesterase 3B
PI3K: phosphatidylinositol 3‐kinase
PKA: protein kinase A
PLIN1: Perilipin‐1
qPCR: quantitative polymerase chain reaction
RNA: ribonucleic acid
Tg: transgene
TG: triglyceride
TGF‐β: transforming growth factor‐beta
TGF‐β: Transforming growth factor‐β
WAT: white adipose tissue
WT: wild type

## 1 INTRODUCTION

Glucose and lipid metabolism disorders are characterized by abnormal blood glucose and lipid levels, which are accompanied by significant pathologies and dysfunctions in metabolic organs. White adipose tissue plays a crucial role in systemic glucolipid metabolism. During times of energy demand, adipocytes release fatty acids through lipolysis to peripheral tissues. However, in individuals with obesity where the insulin/phosphatidylinositol 3‐kinase (PI3K)‐protein kinase B (AKT) signaling is impaired, adipose tissue fails to respond effectively to insulin, resulting in elevated levels of circulating non‐esterified fatty acids (NEFAs) and subsequent metabolic diseases associated with obesity.^1^, ^2^, ^3^ Furthermore, the suppression of glucose transporters in white adipose tissue indirectly leads to insulin resistance in the liver and skeletal muscles.^4^, ^5^ These findings indicate that increased lipolysis and decreased glucose uptake in unhealthy adipose tissue contribute to a global metabolic disorder.

Lipodystrophy is characterized by localized or generalized subcutaneous fat deficiency,^6^ accompanied by a systemic metabolic syndrome. Extensive studies involving clinical cases and animal models of lipodystrophy have contributed to recognizing white adipose tissue as a metabolic regulatory organ.^7^ On one hand, the absence of lipid storage organs leads to the ectopic deposition of lipids in peripheral metabolic organs.^8^ On the other hand, the lack of circulating leptin/adiponectin is associated with diabetes and metabolic complications.^9^, ^10^ In recent years, the increasing use of nucleoside reverse transcriptase inhibitors in patients with HIV has led to lipodystrophy moving away from its previous classification as an orphan disease, posing a threat to the health of a growing number of patients.^11^, ^12^ Clinical studies have revealed significant alterations in the expression of genes encoding PPARγ, CIDEC, perilipin‐1, and AKT‐2 in the adipose tissue of individuals with lipodystrophy.^13^, ^14^, ^15^ However, the underlying mechanisms contributing to metabolic dysregulation in tandem with lipodystrophy are yet to be discovered.

Elevated levels of TGF‐β have been found to be associated with dysfunctional adipose tissue in both human and mouse studies.^16^, ^17^, ^18^, ^19^, ^20^ Adipose tissue obtained from patients with lipodystrophy caused by mutations in the genes encoding DNA polymerase δ and Lamin A/C, as well as HIV infection, has been reported to exhibit increased expression of TGF‐β.^21^, ^22^, ^23^Mechanistic studies focusing on adipose tissue and TGF‐β have primarily investigated its regulatory role in adipose differentiation under physiological conditions.^24^, ^25^ However, the understanding of its regulatory function in diseased adipose tissue remains a subject of debate. First, despite its role as an inhibitor of adipogenesis, the high expression of TGF‐β in obese adipose tissue suggests a potential paradoxical metabolic regulatory role.^16^, ^26^ Second, HIV patients with elevated TGF‐β expression also exhibited higher levels of TNF‐a expression, indicating the existence of a negative feedback system between pro‐ and anti‐inflammatory factors.^22^ These contradictory findings raise a new question: Will the suppression of TGF‐β signaling improve or exacerbate the metabolic status of lipodystrophy?

Based on the holistic and dialectical perspective of Chinese medicine, our focus has been on identifying the common mechanisms and interrelationships contributing to dysregulated glucose and lipid metabolism.^27^ To examine the regulatory effects of TGF‐β inhibition on the lipid and glucose metabolic functions of atrophied adipose tissue, we utilized a lipodystrophy animal model, ap2‐SREBP‐1c mice,^28^ and administered in vivo injections of the small molecule compound SB431542 to inhibit TGF‐β. We collected data on physical indicators and measured the circulating levels of glycolipid metabolites. Subsequently, we assessed the pathological condition of subcutaneous white fat and determined the expression levels of key functional proteins. We also investigated the extent of phosphorylation activation in the upstream AKT signaling pathway. In addition, we employed ob/ob mice, a non‐adipose atrophic glucolipid metabolism disease animal model, to investigate whether TGF‐β exhibits common or specific metabolic regulation in distinct glucolipid metabolism disorder scenarios. Finally, we evaluated the impact of TGF‐β on glucolipid metabolism levels in mature adipocytes in vitro using insulin cocktail reagent‐induced adipogenic 3T3‐L1 cells.

## 2 MATERIALS AND METHODS

### 2.1 Animal

Transgenic aP2‐SREBP‐1c mice in C57BL/6N background were obtained from the Jackson Laboratory (Bar Harbor, ME), breeding with wild type (WT) C57BL/6J females to avoid potential consequences of maternal diabetes; offspring were genotyped by PCR following established protocols. Sixteen‐week‐old male aP2‐SREBP‐1c transgenic mice (Tg) and their homosexual littermates (WT) were used in this study. ob/ob mice in C57BL/6N background were obtained from the Model Animal Research Center of Nanjing University (Nanjing, Jiangsu). Sixteen‐week‐old male ob/ob mutant mice (ob) and their littermates (WT) were used in this study. Mice were housed in a climate‐controlled facility (24°C, 12‐h light/dark cycle, 40%–60% relative humidity) in specific pathogen‐free conditions. The animal care and study protocols were maintained in accordance with the provisions and general recommendations of the Chinese Regulations for the Administration of Affairs Concerning Experimental Animals at Guangdong Pharmaceutical University.

Control group of aP2‐SREBP‐1c and ob/ob wild‐type mice (WT‐veh), group of aP2‐SREBP‐1c and ob/ob wild‐type mice treated with SB431542 (WT‐SB), control group of aP2‐SREBP‐1c transgenic mice (aP2‐veh), group of aP2‐SREBP‐1c transgenic mice treated with SB431542 (aP2‐SB), control group of ob/ob mutant mice (ob‐veh), group of ob/ob mutant mice treated with SB431542 (ob‐SB). The animals were randomized to each group by Excel RAND function.

### 2.2 Oral glucose tolerance test and body composition measurements

GTTs were performed on mice that were fasted for 12 h before measurements. Each mouse received an i.p. injection of glucose (Sigma‐Aldrich, St. Louis, MO, USA) diluted in sterile PBS for a final dose of 2 g/kg body weight. Blood glucose measurements were performed at baseline and after 15, 30, 45, 60, 90, 120, and 150 min. The area under the curve (AUC) was calculated for all groups after each test. Body composition was measured in no anesthetized mice using an LF90II NMR analyzer (Burker, Germany).

### 2.3 RNA extraction and qRT‐PCR analysis

Inguinal white adipose tissue and 3T3‐L1 cells were homogenized in TAKARA RNAisoPlus reagent (Takara), and single standard complimentary deoxyribonucleic acid (cDNA) was synthesized by using Prim eScript RT Reagent Kit (Takara, USA). Quantitative real‐time PCR was performed with Thermo Scientific PikoReal 96 Real‐Time PCR System (Waltham, USA). Each reaction was performed in duplicate and the value of the gene of interest was normalized to mouse GAPDH or RPS18 expression. The comparative threshold cycle (CT) method was used to calculate the relative expression. The specific primers are shown in Table S1.

### 2.4 Western blot

Differentiated cells were washed with ice‐cold phosphate buffed saline (PBS) and were lysed in RIPA Cell Lysis Solution. Insoluble protein was removed by centrifugation at 12000 ***g*** for 30 min and soluble protein concentrations were measured by using a BCA protein assay kit (Biotime, Shanghai, China). Equal amounts of protein were resolved by 10% SDS–polyacrylamide gel electrophoreses (SDS–PAGE) and transferred to polyvinylidene difluoride membranes (Millipore, Darmstadt, Germany), and hybridized with primary anti‐p‐Smad3(phospho S423 + S425) (1: 2000, ab52903, Abcam), anti‐p‐AKT1(phospho S473) (1:1000, ab81283, Abcam), anti‐p‐AKT2(phospho S474) (1:1000, ab38513, Abcam) anti‐GAPDH (1:1000, ab181602, Abcam), antibodies overnight at 4°C. After incubated with a 1:10000 dilution of horseradish peroxidase‐conjugated secondary antibody for 2 h at room temperature, protein bands were visualized with SuperSignal West Femto Maximum Sensitivity Substrate Kit (Thermo Fisher Scientific, Rockford, USA) and captured by using an MiniChemi610 imager (Sage Creation Science, Beijing, China).

### 2.5 Cell counting kit‐8 (CCK8) assay

CCK8 assay was conducted to evaluate the cytotoxicity. The cell suspension was prepared and seeded into the 96‐well plate (5000 cells/well) after treatment. Then, the cells were treated with different concentrations of SB431542 (4‐[4‐(2H‐1,3‐Benzodioxol‐5‐yl)‐5‐(pyridin‐2‐yl)‐1H‐imidazol‐2‐yl] benzamide) (Selleck, USA) and conventional cultured in a carbon dioxide incubator for 4 h. After adding CCK8 reagent in one‐tenth of the culture medium volume to each well, the cells were continued to be incubated for another 4 h. The OD value at 450 nm was detected utilizing a microplate reader. The curve was drawn using GraphPad Prism 5.0.

### 2.6 Cell culture, adipogenesis differentiation, and SB431542 treatment

3T3‐L1 preadipocytes were cultured in DMEM supplemented with 10% FBS (Gibco, USA) and 1% antibiotics (Gibco, USA) at 37°C and 5% CO_2_. The old medium is replaced with a new medium every 2 days. After reaching confluence around 80%, cells were treated with differentiation cocktails including 0.5 mM dexamethasone (SIGMA, USA), 0.5 mM 3‐isobutyl‐1‐methylxanthine (IBMX) (SIGMA, USA), 2 mM troglitazone (SIGMA, USA), and 1.7 mM insulin (Biosharp, Hefei, China) for 48 h, followed by 1.7 mM insulin treatment for 4–6 days. Then, total protein and mRNA were extracted for further analysis. For the SB431542 dosing group (SB‐treated), the final dosing concentration was determined according to CCK‐8 results. TGF‐β inhibitor was administered for 6 h per day, and the blank control group (control) was cultured daily with a normal medium. After 2 days, we evaluated the degree of cell differentiation into mature adipocytes and extracted total protein and total RNA.

### 2.7 SB431542 treatment for mice

SB431542 was initially dissolved in DMSO, aided with 30% PEG200, and then dissolved in normal saline. Mice were injected intraperitoneally with a dose of 10 mg/kg (BW), and the control group was injected with the same dose of solvent. Doses were given daily for 2 weeks.

### 2.8 Statistics

Differences between every two groups were analyzed using Student's *t*‐test. Differences were considered statistically significant at *p* < .05. Statistics were performed using Stata 17 (StataCorp LP, College Station, Texas, USA).

### 2.9 Target and Ligand Details

The protein targets and ligands discussed in this article are linked to their respective entries on https://www.guidetopharmacology.org, the central repository for data sourced from the IUPHAR/BPS Guide to PHARMACOLOGY. These links ensure seamless access to detailed information. In addition, for long‐term accessibility, the information is permanently archived in the Concise Guide to THE CONCISE GUIDE TO PHARMACOLOGY 2021/22: Catalytic receptors.^29^

## 3 RESULTS

### 3.1 Extreme dysfunctional adipose tissue exhibits upgraded TGF‐β signaling and Disturbed glucose metabolism

We observed both aP2‐SREBP‐1c mice and ob/ob mice exhibit glucose metabolism disorder evidenced by reduced glucose tolerance (Figure S1A–D). Analysis of mRNA transcript levels revealed that their iWAT both represented enhanced TGF‐β signaling pathways (Figure S2A,C), which plays an essential role in limiting hyperplastic expansion and remodeling.^30^ These results illustrate that either lipodystrophy or hypertrophy showed Glucose metabolism disorder which is related to upregulated TGF‐β signaling.

### 3.2 Block of the TGF‐β pathway alleviates ectopic lipid storageinaP2‐SREBP‐1c mice, not ob/ob mice

**FIGURE 1:**
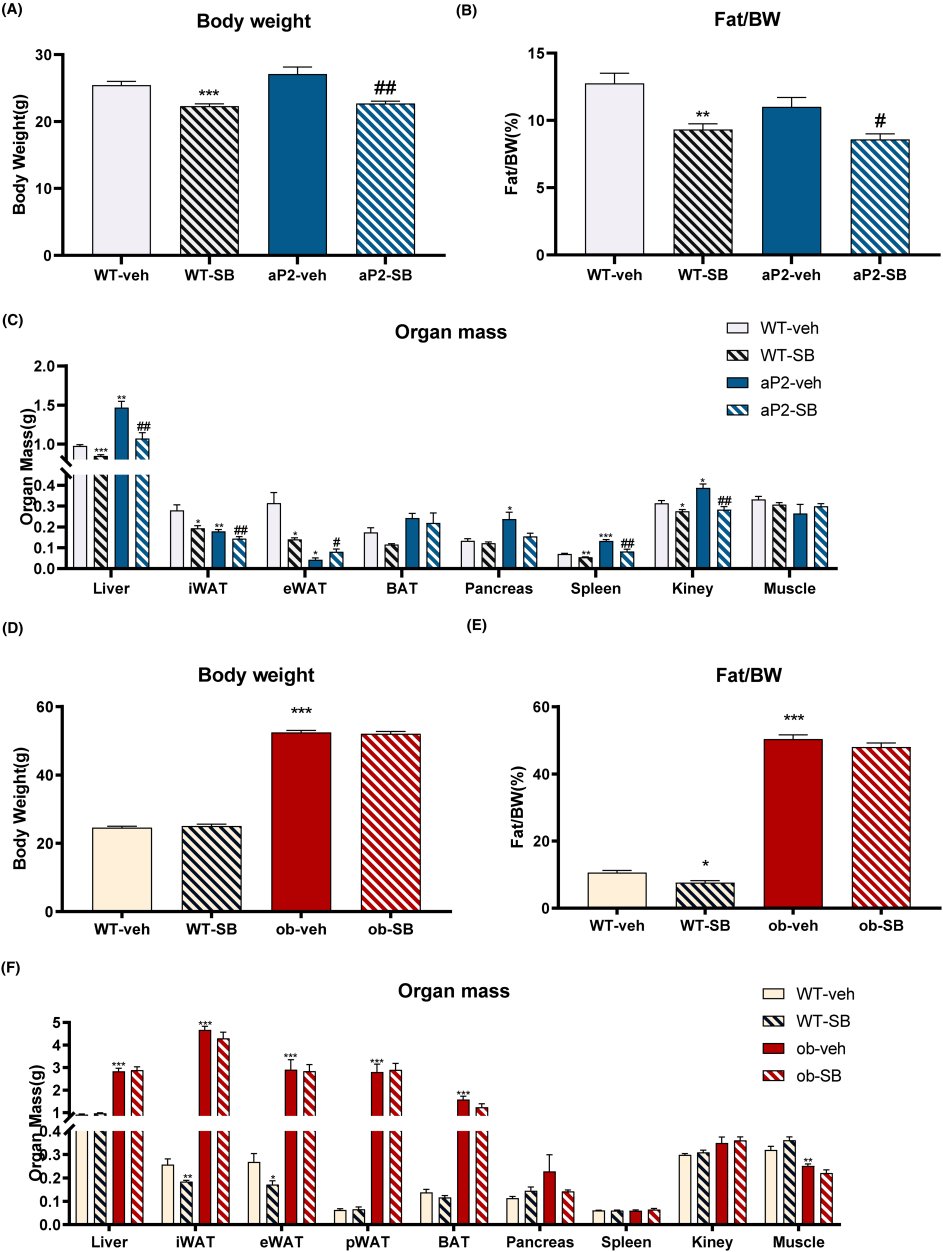
Effects of TGF‐β inhibitor on body weight, fat/body weight ratio, and organ mass of aP2‐SREBP‐1c mice and ob/ob mice. Changes in body weight of aP2‐SREBP‐1c mice during treatment (A), Fat to body weight ratio (B), Organ weight (C). Changes in body weight of ob/ob mice during treatment (D), Fat to body weight ratio (E), Organ weight (F). Compared with the WT‐veh group, *p < .05, **p < .01, ***p < .001. Compared with the aP2‐veh/ob‐veh group, #*p* < .05, ##*p* < .01, ###*p* < .001.

To demonstrate the association between TGF‐β and fat storage function, we pretreated aP2‐SREBP‐1c mice and ob/ob mice with SB431542, the TGF‐β type I receptor inhibitor. We observed aP2‐SREBP‐1c and wild‐type mice with SB431542 administration, gained less weight (Figure 1A) and lower fat mass to weight ratio (Figure 1B). Elevated white fat weight in the epididymis was accompanied by a decrease in the liver, kidney, and spleen (Figure 1C), which suggests improved WAT lipid storage capacity and lipid ectopic deposition (no Statistical Difference of these peripheral organs between WT‐veh and aP2‐SB groups). By comparison, we observed ob/ob mice hardly changed in body weight (Figure 1D), fat mass to weight ratio (Figure 1E), and organ mass (Figure 1F).

**FIGURE 2:**
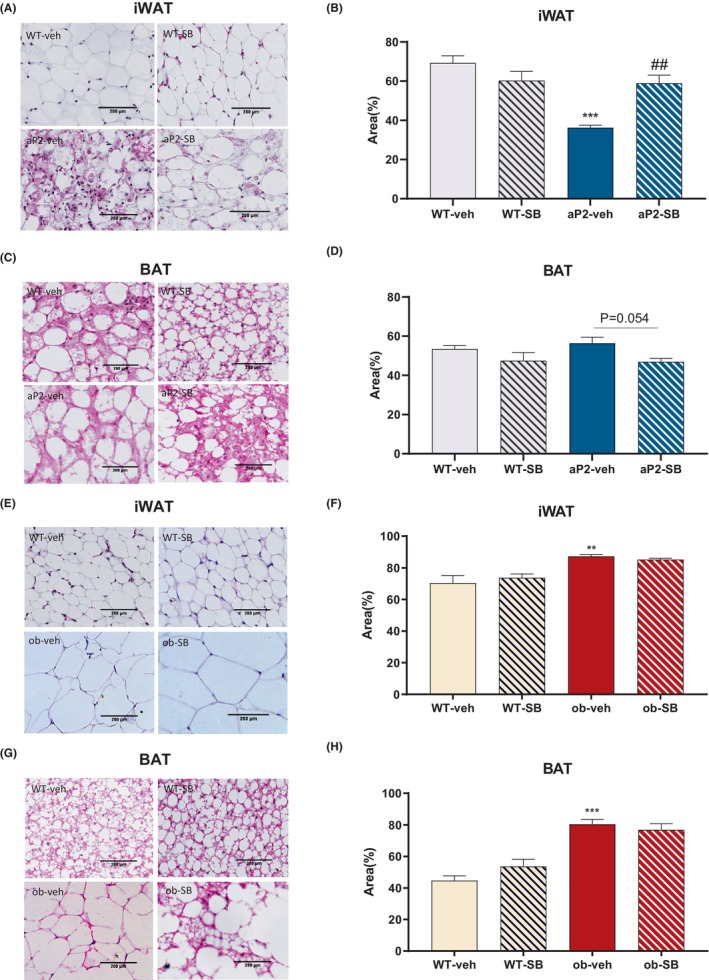
Hematoxylin–Eosin (H&E) stained sections of iWAT in aP2‐SREBP‐1c mice and ob/ob mice. HE staining of iWAT and area of lipid droplet in aP2‐SREBP‐1c mice (A, B) and ob/ob mice (E, F). HE staining of BAT and area of lipid droplet in aP2‐SREBP‐1c mice (C, D) and ob/ob mice (G, H), the pathological section shown here was shot at 20 × scope (*n* = 3). Compared with the WT‐veh group, *p < .05, **p < .01, ***p < .001. Compared with the aP2‐veh/ob‐vehgroup, #*p* < .05, ##*p* < .01, ###*p* < .001.

We further examined the AT compartment in greater detail and found that iWAT expanded markedly and represented a unilocular lipid droplet (Figure 2A,B), and brown adipose tissue (BAT) shrunk slightly and represented multilocular lipid droplet by contrary (Figure 2C,D), which demonstrated improvement of ectopic lipid storage. However, iWAT hypertrophy and BAT whitening had no significant reverse in ob/ob mice (Figure 2E–H). These findings suggest that blocking the TGF‐β pathway has a positive impact on the structure and Lipid loading capacity of waste adipose tissue, while inhibitor has little interference on a relative hypertrophy adipose tissue.

### 3.3 Block of the TGF‐β pathway alleviates abnormal glucose and lipid metabolism of aP2‐SREBP‐1c mice not ob/ob mice

**FIGURE 3:**
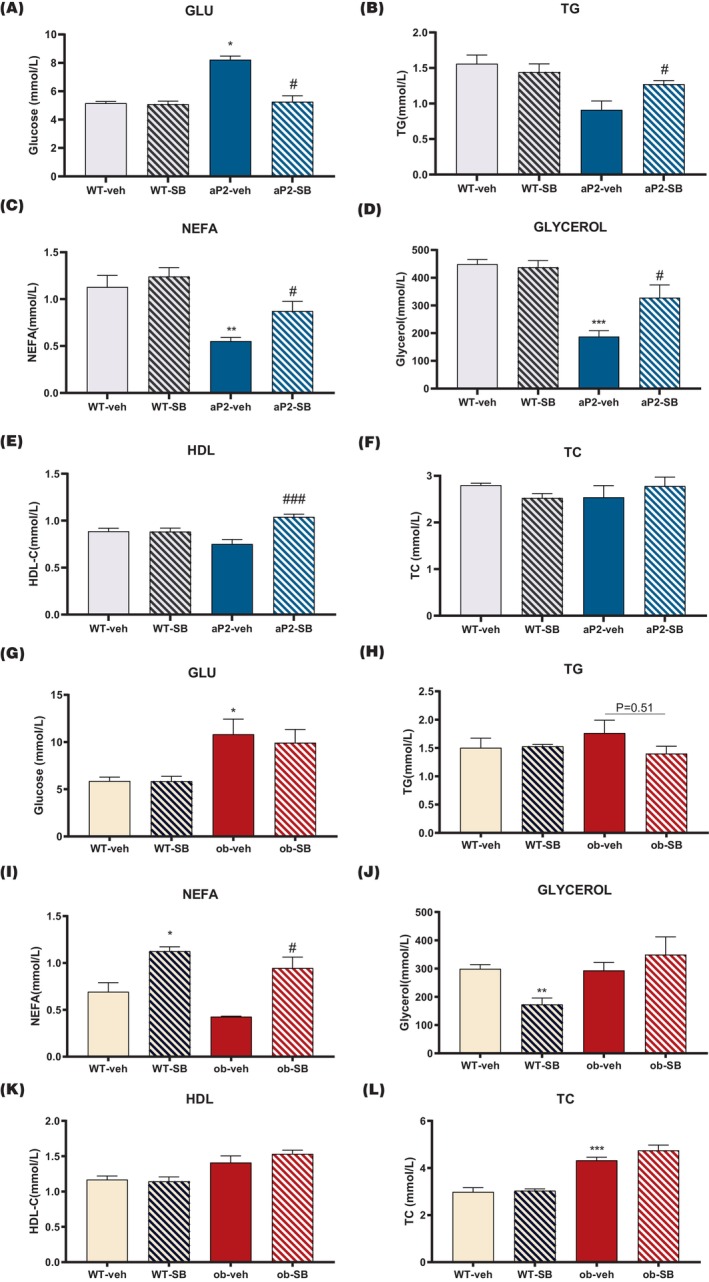
Effects of TGF‐β inhibitor on abnormal Glucolipid metabolism in aP2‐SREBP‐1c mice and ob/ob mice. The fasted plasma Glu (A), TG (B), NEFA (C), Glycerol (D), HDL (E), and TC (F) of aP2‐SREBP‐1c mice and their littermates with or without SB431542 treatment. The fasted plasma Glu (G), TG (H), NEFA (I), Glycerol (J), HDL (K), and TC (L) of ob/ob mice and their littermates with or without SB431542 treatment (*n* = 4–5 per group). Compared with the WT‐veh group, *p < .05, **p < .01, ***p < .001. Compared with the aP2‐veh/ob‐veh group, #*p* < .05, ##*p* < .01, ###*p* < .001.

As mentioned above, iWAT functional status affects the systemic disorders of glucolipid metabolism. Consequently, we compared glucose levels and lipid metabolism in aP2‐SREBP‐1c mice and ob/ob mice. First, aP2‐SREBP‐1c mice treated with SB431542 displayed reduced glucose levels (Figure 3A) and enhanced TG (Figure 3B), NEFA (Figure 3C), glycerol (Figure 3D), and high‐density lipoprotein (HDL) (Figure 3E), while total cholesterol (TC) levels remained unchanged (Figure 3F). These findings suggest that the circulating glucose and fat levels gradually return to physiological values.

On the contrary, glucolipid metabolism desorders of ob/ob mice were slightly influenced by SB431542 as evidenced by no significant difference in GLUT, TG, glycerol, HDL, TC levels (Figure 3G–H, J–L) and increased NEFA (Figure 3I). In brief, transforming growth factor‐beta inhibitor improves hyperglycemia and dyslipidemia in aP2‐SREBP‐1c mice, not ob/ob mice.

### 3.4 Block of the TGF‐β pathway partially upregulates mRNA expression of lipases and GLUTs in dysfunctional adipose tissue

**FIGURE 4:**
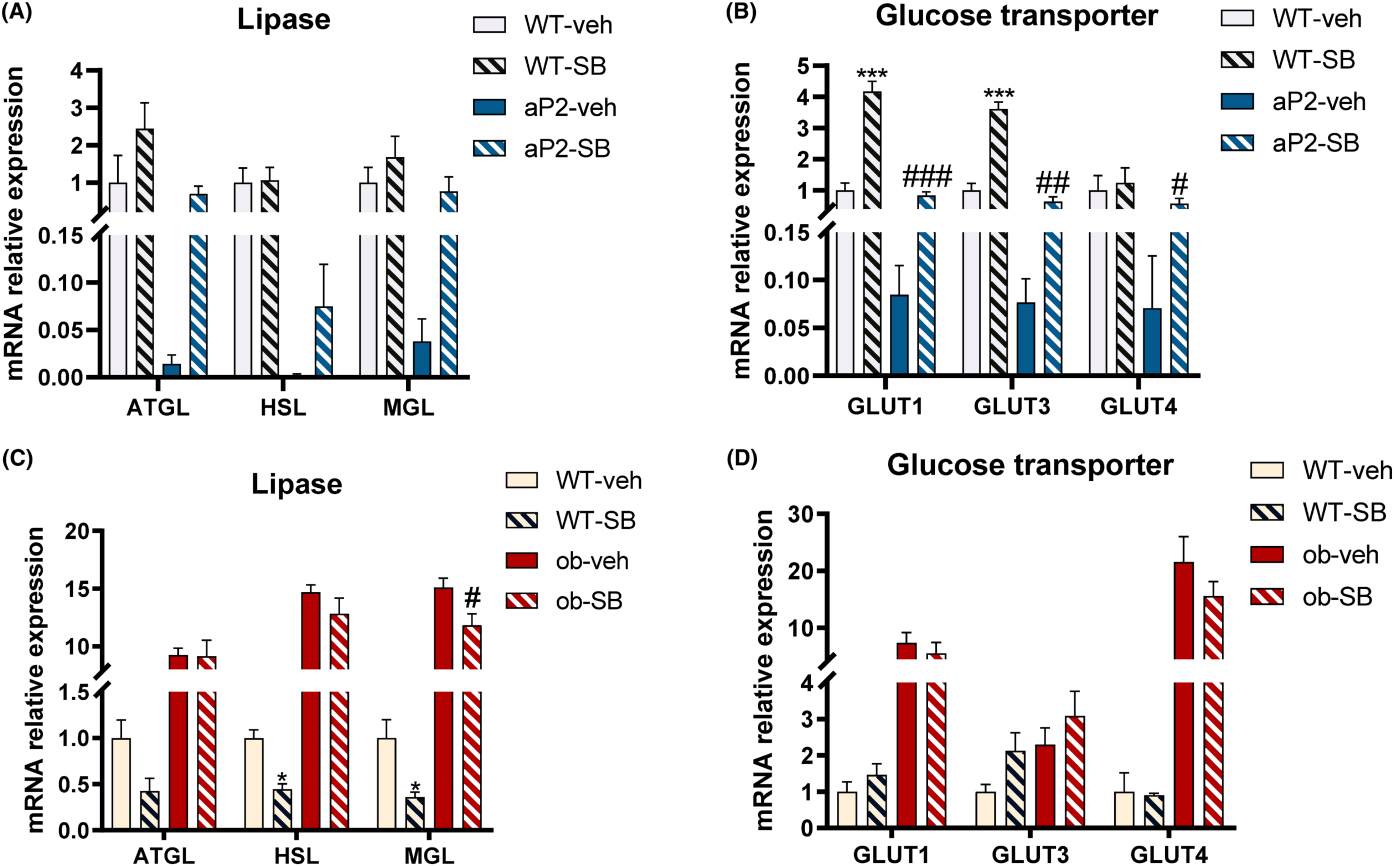
Effects of TGF‐β inhibitor on lipolytic and glucose transporters related genes in aP2‐SREBP‐1c mice and ob/ob mice. Related RNA levels of lipolytic related lipases (A, C) and glucose transporters (B, D), were determined in iWAT (*n* = 4–5). Compared with the WT‐veh group, *p < .05, **p < .01, ***p < .001. Compared with the aP2‐veh/ob‐veh group, #*p* < .05, ##*p* < .01, ###*p* < .001.

In the iWAT of mice in the aP2‐SB group compared to the aP2‐veh group, mRNA levels of the TGF‐β signaling pathway were significantly reduced (Figure S2A) and lipolysis‐related genes were increased (Figure 4A), although the latter was not significantly different. Expectedly, mRNA levels of glucose transporters (GLUT1, GLUT3, and GLUT4), expressing with low levels in ap2‐veh mice, were markedly restored to WT‐veh group level in iWAT of mice in aP2‐SB group (Figure 4B) (no Statistical Difference of these peripheral organs between WT‐veh and aP2‐SB groups). After TGF‐β blocker treatment in the iWAT of ob/ob mice, mRNA levels of TGF‐β signaling pathway (Figure S2C) were also significantly reduced, but only MGL was significantly reduced in lipolysis‐related genes (Figure 4C). Similarly, there were no significant changes in the transcript levels of GLUTs (Figure 4D) in the iWAT of ob/ob mice (no statistical significance). These results indicate that blockage of TGF‐β signaling partially improves key functional protein gene expression.

### 3.5 TGF‐β inhibitor improves the release of TG and glycerol of 3T3‐L1 cells

**FIGURE 5:**
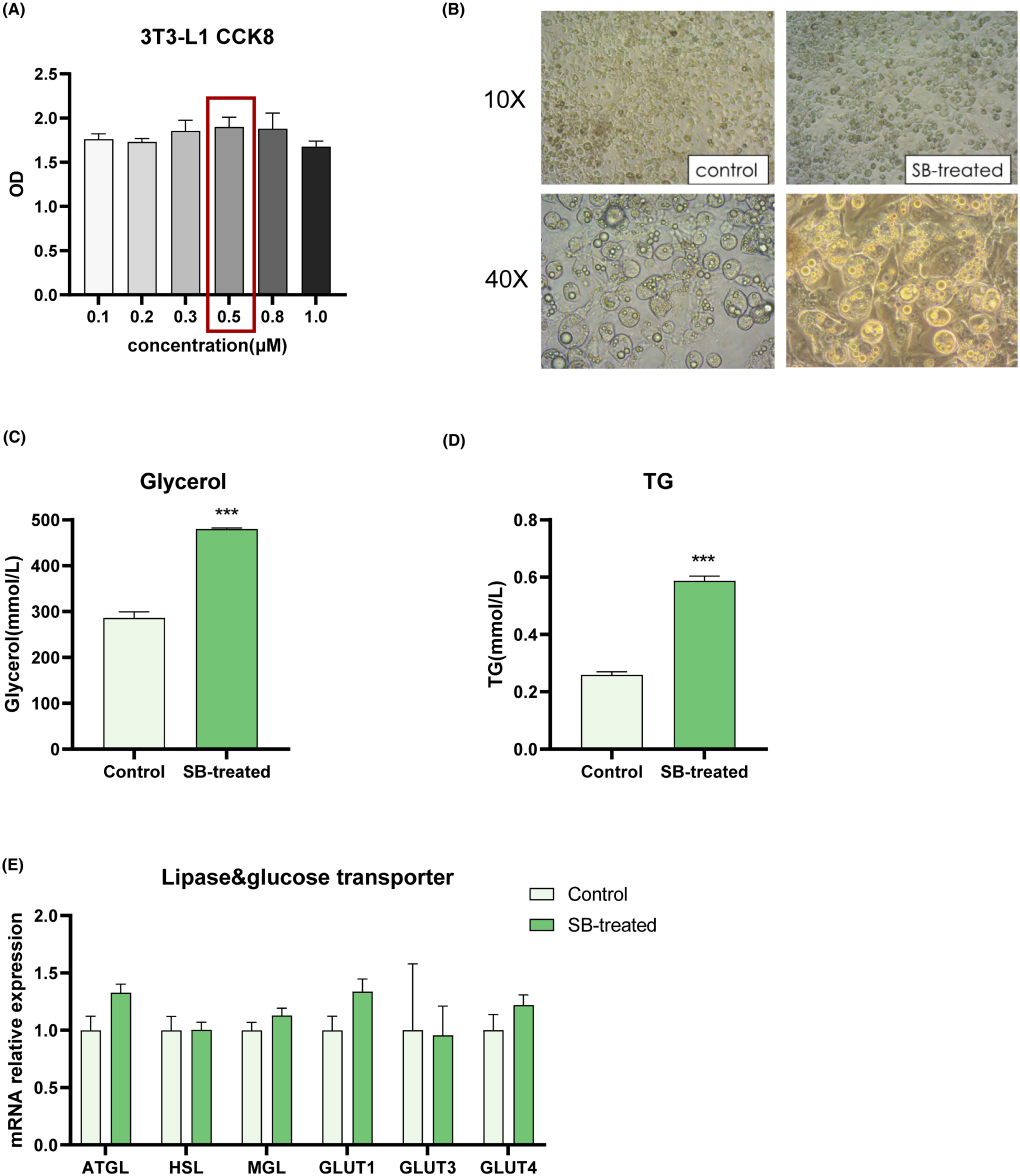
Effects of TGF‐β inhibitor on Glucolipid levels, glucose transporters, and lipolysis‐related genes of 3T3‐L1 mature adipocyte in vitro. The concentration of SB431542 in 3T3‐L1 mouse embryonic fibroblasts (A). Cell morphology under microscope after lipid formation induction which were captured at 10 × scope and 40 × scope by Inverted microscope (B). The Glycerol (C), TG (D) in the cell supernatant with or without SB431542 treatment. The mRNA level of lipolytic‐related lipases and glucose transporters (E) of 3T3‐L1 mature adipocytes with or without SB431542 treatment (*n* = 3). Compared with the control group, *p < .05, **p < .01, ***p < .001.

Complex pathological states of the in vivo environment play critical roles in iWAT metabolism. We used in vitro 3T3‐L1 cell experiment to verify that TGF‐β inhibitor improves the lipogenesis. First, we figured out the appropriate concentration of the inhibitor according to the results of the CCK8 assay (Figure 5A). After inducing adipogenesis and treating 3T3‐L1 cells with or without SB431542, we observed the differentiation of cells into mature adipocytes (Figure 5B). Interestingly, the levels of TG and glycerol in the supernatant were significantly elevated, indicating enhanced lipogenesis (Figure 5C,D). But lipolytic enzymes related gene expression have no difference. These results illustrate that TGF‐β inhibitor confers increased lipogenesis independent of the expression of lipolytic enzymes. Furthermore, the absence of alterations in the transcript levels of GLUT‐related genes underscore that TGF‐β treatment exerts no discernible effects on glucose transport (Figure 5E).

### 3.6 Block of the TGF‐β pathway activates insulin downstream AKT pathway in dysfunctional adipose tissue

The aforementioned findings highlight the impact of a TGF‐β inhibitor on the glycolipid metabolism of adipocytes in different physiological and pathological states. While the expression levels of GLUTs and lipases are influenced by the activation status of AKT signaling downstream of insulin. In line with expectations, treatment with SB431542 resulted in a significant upregulation of mRNA levels of critical genes in PI3K/AKT pathways in the iWAT of aP2‐SREBP‐1c mice (Figure S2B). In contrast, these genes showed a partial decrease (Figure S2D) in the iWAT of ob‐SB group mice. Furthermore, protein levels of p‐Smad3 were significantly reduced (Figure S3A,B) in the iWAT of aP2‐SB group mice, confirming the blockade of TGF‐β signaling by SB431542. Moreover, p‐AKT1 and p‐AKT2 (Figure S3A–D) were significantly increased, which is required for the insulin regulation of the pathways that control systemic glucose homeostasis. These results illustrate that suppression of TGF‐β signaling confers prevented ectopic fat storage and maintains homeostasis of glycolipid metabolism through activating AKT in aP2‐SREBP‐1c mice. Similarly, in the inguinal white adipose tissue (iWAT) of ob‐SB group mice, we observed that the inhibition of TGF‐β led to a significant increase in the protein levels of p‐AKT1 and p‐AKT2 (Figure S3E–H). However, the improvement in abnormal blood glucose and lipids achieved by TGF‐β inhibition is limited in extremely obese conditions. The outcome of this conflict requires further discussion.

## 4 DISCUSSION

During the development of lipodystrophy, the TGF‐β/Smad3 pathway contributes to the impaired functionality of adipose tissue in nutrient storage.^23^ However, the impact of TGF‐β inhibition on the abnormal state of glucolipid metabolism caused by fat loss remains unclear. Our findings demonstrate that inhibiting TGF‐β signaling enhances the size of subcutaneous white adipocytes and improves hyperglycemia and dyslipidemia in aP2‐SREBP‐1c mice. Furthermore, we validated the specific role of TGF‐β in regulating the metabolic status of distinct diseased inguinal white adipose tissue (iWAT) by utilizing the ob/ob animal model. Interestingly, aP2‐SREBP‐1c mice exhibit an elevated glucose set point, while the shape of the glucose curve (not the area under the curve) appears similar, whereas ob/ob mice maintain normal glycemia but display impaired glucose tolerance. We speculate that ob/ob mice possess a sufficient number of mature adipocytes and high expression of insulin‐related genes, allowing them to maintain lower blood glucose levels in a fasting state.^31^, ^32^, ^33^ However, their glucose tolerance significantly decreases with increasing insulin concentration.

In aP2‐SREBP‐1c mice, we observed improved lipid ectopic deposition followed by expansion of inguinal white adipose tissue (iWAT). In contrast, the morphology of white inguinal fat in ob/ob mice was not significantly altered. These results suggest that blocking TGF‐β may restore the differentiation of preadipocytes into mature adipocytes. Despite the deficiency in mature adiposity observed in aP2‐SREBP‐1c mice, the high level of pref‐1 expression indicates substantial recruitment of preadipocytes.^28^ Previous studies have shown that TGF‐β exerts an inhibitory effect on adipocyte differentiation.^34^ The increasing trend in the expression levels of GLUTs and lipase in adipose tissue after TGF‐β inhibitor injection also suggested that the atrophied adipose tissue gradually acquired the metabolic regulation function of mature adipose tissue. However, ob/ob mice, as a typical representative of diet‐induced obesity, have low levels of preadipocyte markers^35^ in white adipose tissue and lack the function of recruiting adipose progenitor cells.^36^ Correspondingly, there were no significant changes in GLUTs and lipase in the adipose tissue of ob/ob mice. Therefore, after blocking the TGF‐β‐Smad3 signaling axis, the two adipose pathological states showed differences in metabolic functional changes.

Analysis of the adipose function alterations mentioned above. Adipogenesis, lipolysis, and glucose transport are all influenced by the activation status of AKT signaling downstream of insulin.^37^ AKT signaling was significantly activated in the adipose tissue of aP2‐SREBP‐1c mice, suggesting an activating role of the TGF‐β‐AKT axis for atrophic adipose tissue function. Paradoxically, activated AKT signaling in the adipose tissue of ob/ob mice is uncoupled from metabolic recovery, which may stem from a severe impairment in metabolic flexibility.^38^ On one hand, both GLUTs and insulin transcript levels are elevated in ob/ob mice compared to the control group, indicating that GLUTs protein expression is already impaired in long‐term obesity.^39^ Recent research has shown that inhibition of AKT phosphorylation does not eliminate insulin‐mediated anti‐lipolysis,^40^, ^41^ which may explain the dissociation between insulin signaling activation and its anti‐lipolytic effects. On the other hand, when compared with aP2‐SREBP1c mice, ob/ob mice exhibit a significantly higher rate of blood glucose increase than the control group after being fed a glucose solution. This suggests that ob/ob mice have a weaker buffering capacity against caloric stress compared to aP2‐SREBP‐1c mice. The systemic metabolic homeostasis is jointly regulated by the liver, muscles, and other organs, and is not solely determined by insulin AKT signaling in adipose tissue.^38^ The abnormally elevated food intake^9^ and circulating levels of triglycerides (TG) and total cholesterol (TC) also indicate that ob/ob mice face more severe metabolic stress challenges in peripheral organs than aP2‐SREBP‐1c mice.

This study also has some shortcomings: The inhibition of TGF‐β lacks organ specificity, so whether the improvement of adipose ectopic deposition is dependent on the functional recovery of adipose tissue is open to question. Under the premise of AKT activation, we observed changes in transcript levels of GLUTs, and lipases, but did not focus on changes in the level of these key proteins and insulin subsequently. By advancing further experiments, we may be able to target the specific links involved in regulating the effects of glucolipid metabolism in fat overload or fat deficiency states.

Insulin resistance (IR) is one of the pathological cores of Glucolipid Metabolic Disorders (GLMDs).^42^ Therapeutic strategy concurrently targeting the impairment in insulin sensitivity according to the holistic view of traditional Chinese medicine has been shown to improve the abnormalities in the metabolism of both glucose and lipid. This study demonstrates that TGF‐β inhibition activates AKT signaling in atrophic and hypertrophic adiposity, ameliorating adipose morphological function and systemic glucolipid metabolic disorders in aP2‐SREBP‐1c lipodystrophy mice without similar effects in ob/ob mice. TGF‐β‐Smad3‐AKT signaling may provide new therapeutic options for disorders of glucolipid metabolism in the context of lipodystrophy. Meanwhile, its functional specificity in different pathological states, and its contribution to metabolism by the major cells from which it is released will be the next direction of interest.

## AUTHOR CONTRIBUTIONS

*Participated in research design*: Guo Jiao and Xianglu Rong. *Conducted experiments*: Wendong Xu, Shuizheng Lai, and Jia Zhao. *Performed data analysis*: Shijie Wei, Xueying Fang, and Yiyi Liu. *Wrote or contributed to the writing of the manuscript*: Wendong Xu, Xianglu Rong, and Shuizheng Lai.

## FUNDING INFORMATION

This work was supported by the Key Project of National Natural Science Foundation of China (81 530 102 and 81 830 113); National key R & D plan “Research on modernization of traditional Chinese medicine” (2018YFC1704200) and Major basic and applied basic research projects of Guangdong Province of China (2019B030302005).

## CONFLICT OF INTEREST

The authors declare that the research was conducted in the absence of any commercial or financial relationships that could be construed as a potential conflict of interest.

## ETHIC STATEMENT

The animal study was reviewed and approved by the Experimental animal ethics committee of Guangdong Pharmaceutical University.

## Supporting information


**Data S1.**Supporting Information.

## Data Availability

The data that support the findings of this study are available from the corresponding author upon reasonable request.
