# Supplementary material for: The blockade of the TGF‐β pathway alleviates abnormal glucose and lipid metabolism of lipodystrophy not obesity
Source: Pharmacol Res Perspect. 2024 Jan 4;12(1):e1160. doi: 10.1002/prp2.1160 (PMC10765454; doi:10.1002/prp2.1160)
Supplement: Supplementary file 1 — Data S1.Supporting Information. [file PRP2-12-e1160-s001.pdf]

Supplementary Figure/Table legends

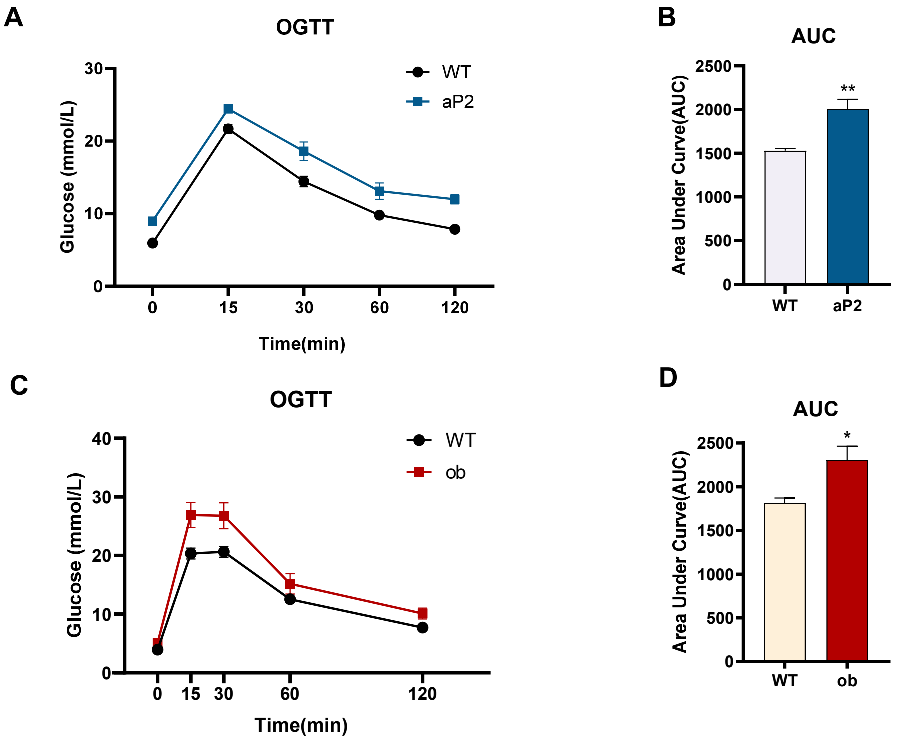

Fig S1. Oral Glucose Tolerance Tests in aP2-SREBP-1c mice and ob/ob mice.

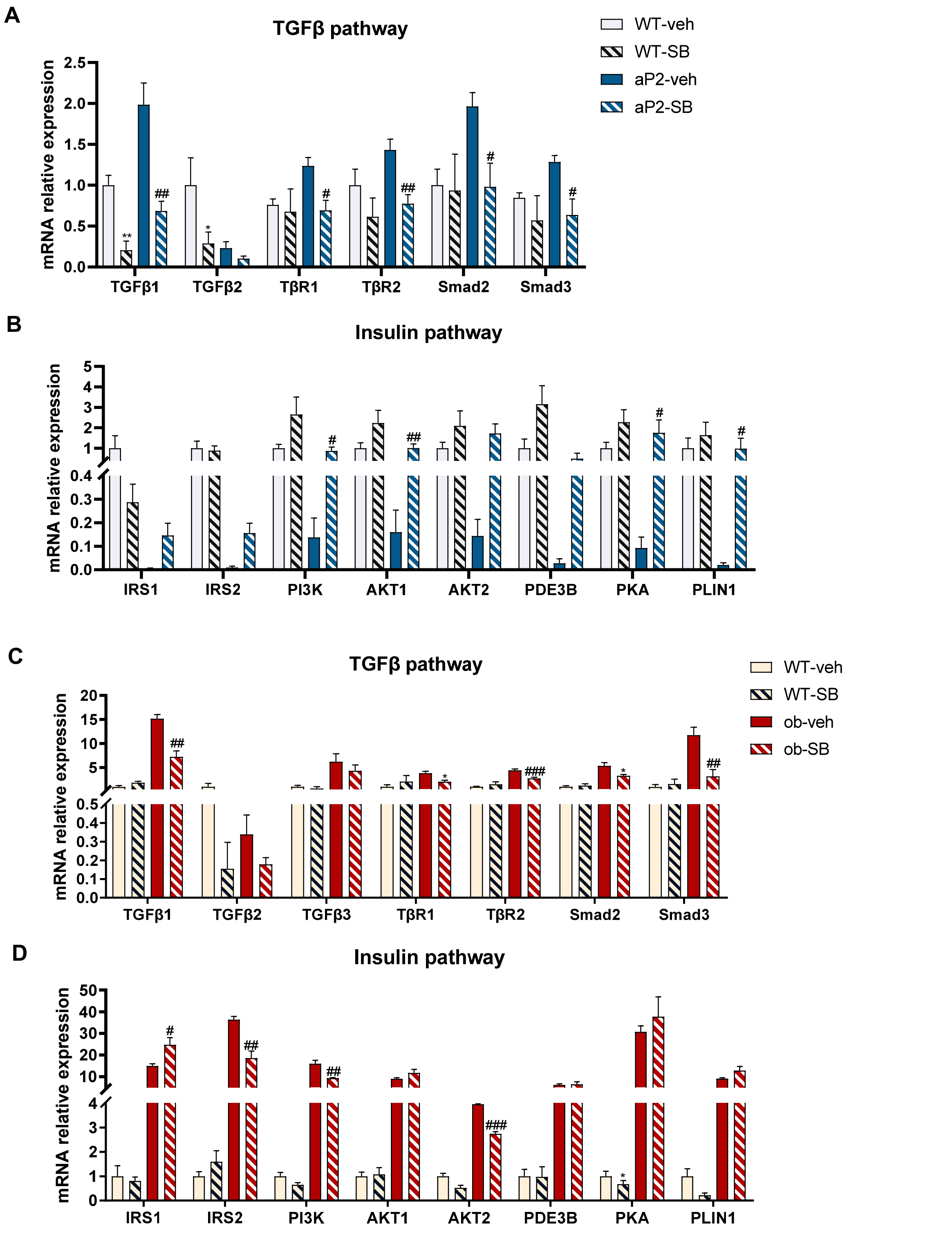

Fig S2. Effects of TGF-β inhibitor on TGF-β and insulin transcription cascade in atrophy and hypertrophy adipose tissue.

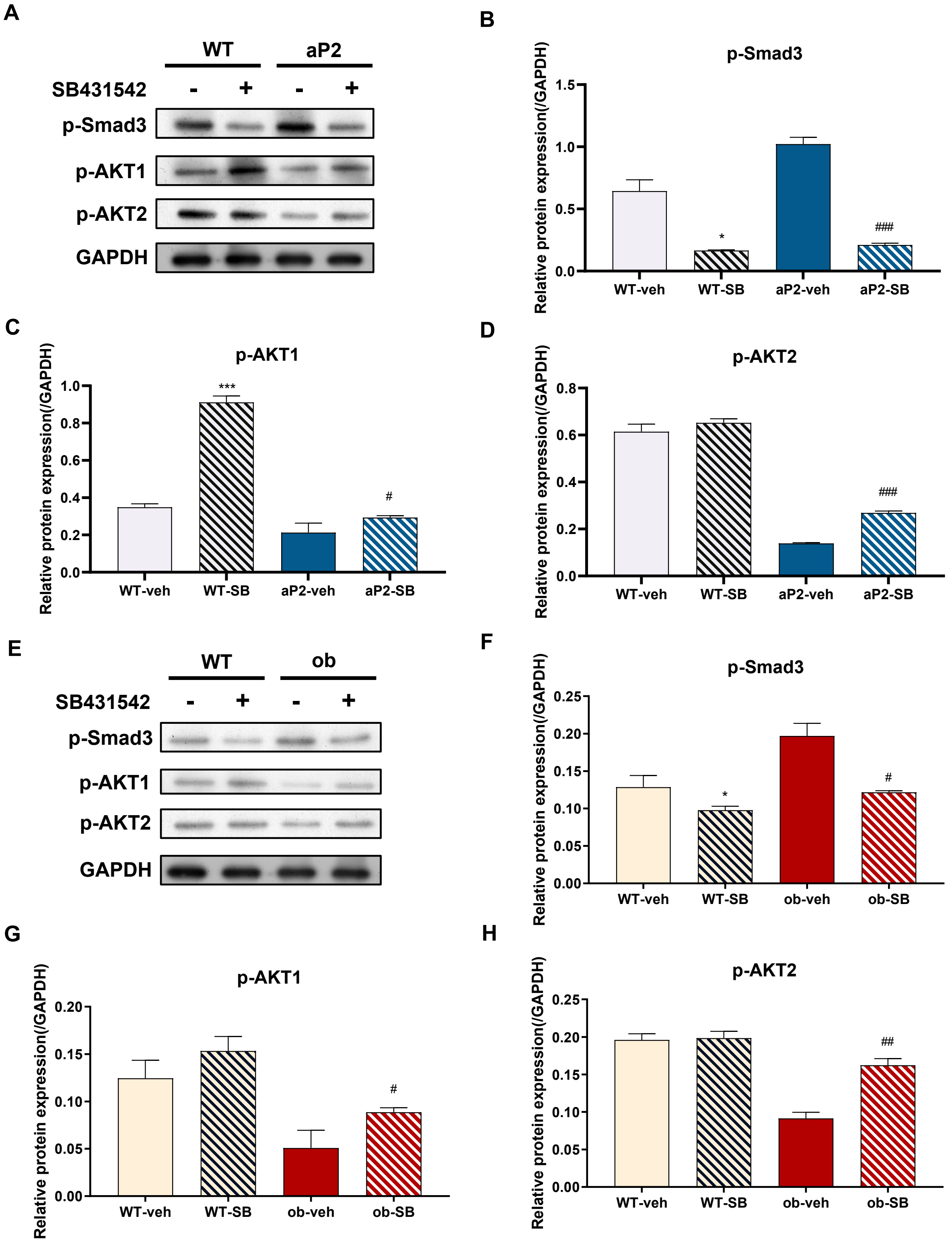

Fig S3. Semi-quantitative analysis of AKT phosphorylation in atrophy and hypertrophy adipose tissue under the treatment of TGF-β inhibitor.

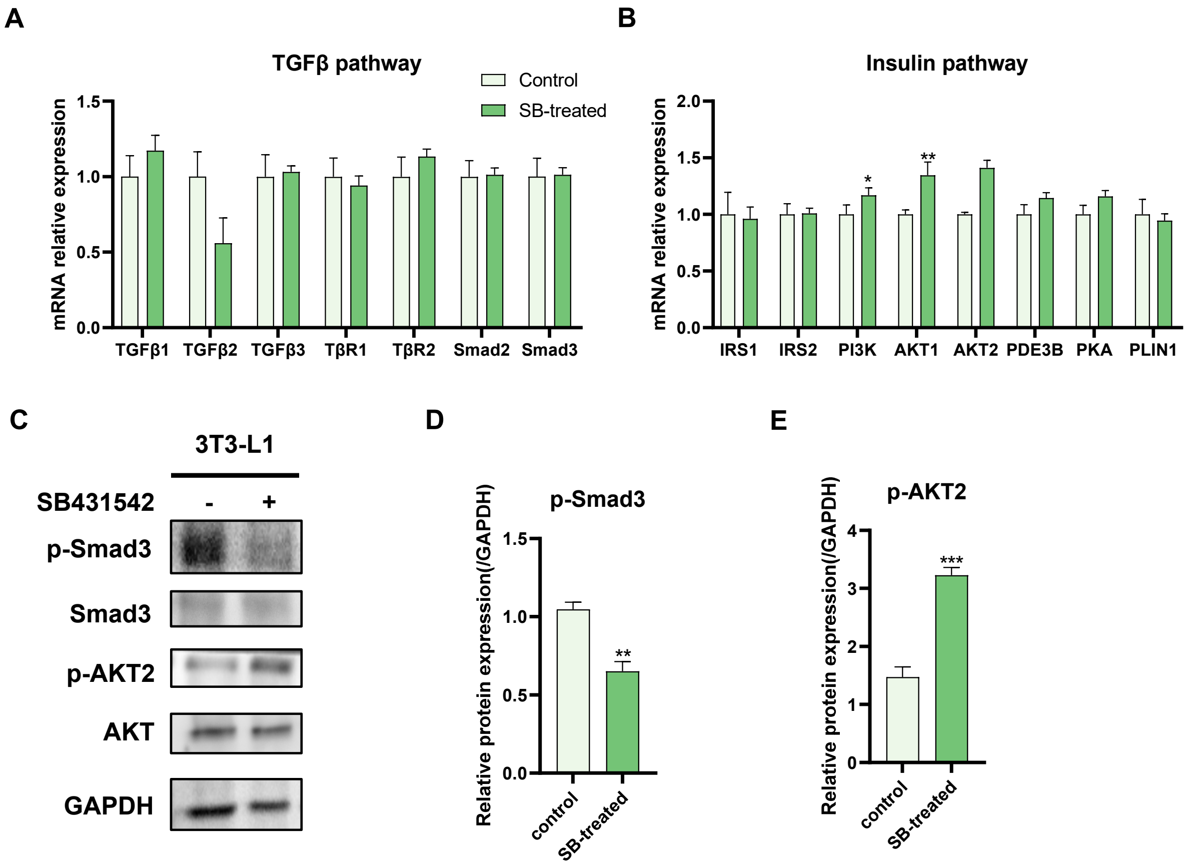

Fig S4. Effects of TGF-β inhibitor on TGF-β and insulin pathway of 3T3-L1 mature adipocyte in vitro.

TableS1. primer sequences for quantitative polymerase chain reactions

Target gene	Forward primer sequence (5'-3')	Reverse primer sequence (5'-3')
Rps18	CATGCAAACCCACGACAGTA	CCTCACGCAGCTTGTTGTCTA
ATGL	CAGAGATGGACTTCGATTCCTT	CAGGTGCTCTAGAATTCGATCT
HSL	GCGCTGGAGGAGTGTTTTT	CCGCTCTCCAGTTGAACC
MGL	TTTTGTCCTGCCAAATATGACC	CAGAGTTGTACAGGTCAACCTC
IRS1	CAAGACGCTCCAGTGAGGATTTAAG	AGACGTGAGGTCCTGGTTGTGA
IRS2	CCAGCATCAGCTGCCACTTC	ACACCAAAGGCCATCTCGGTATAG
PI3K	TTCAACAAAGACGCACTCCTGAAC	GTCACCAATGCCGAGGACATAA
AKT1	TGCACAAACGAGGGGAATATAT	CGTTCCTTGTAGCCAATAAAGG
AKT2	CAAGGTACTTTGATGACGAGTTCAC	CTGCTCACTCTCGGATGCTG
PDE3B	GAAAAAGTGCCTGTGATCAGAC	TCTGTTCTCGGGAAATACAAGG
PKA	CTTCTCAGAATACAGCCCAGTT	TTGTCTAAGATCTTCATGGCGT
PLIN1	GCACAACCTGGCAGCCTCTC	CCTCCTCGTCGTCTGTCTCCTC
TGF-β1	CCAGATCCTGTCCAAACTAAGG	CTCTTTAGCATAGTAGTCCGCT
TGF-β2	CTCGACATGGATCAGTTTATGC	ATAAACCTCCTTGGCGTAGTAC
TGF-β3	ATCAAGAAGAAGAGGGTGGAAG	GTAAAGTGCCAGGACCTGATAG
TβR1	AGCTGGCCTTGGTCCTGTGG	TGGTGAATGACAGTGCGGTTATGG
TβR2	GACCTCAAGAGCTCTAACATCC	GTCATCCACAGACAGAGTAGG
Smad2	GTCGTCCATCTTGCCATTCACTCC	GCTCTCCACCACCTGCTCCTC
Smad3	GTGCGAGAAGGCGGTCAAGAG	CCACAGGCGGCAGTAGATAACG
GLUT1	GAAGAAGGTCACCATCTTGGAG	CGAAGATGCTCGTTGAGTAGTA
GLUT3	TGGGATCAATGCTGTGTTCTAT	CCAGGAACAGAGAAACTACAGT
GLUT4	TATTCAACCAGCATCTTCGAGT	GTCCAGCTCGTTCTACTAAGAG
